# Appropriating the civil sphere: the construction of German collective identity by right-wing populist actors during the Covid-19 pandemic

**DOI:** 10.1057/s41290-023-00189-2

**Published:** 2023-03-04

**Authors:** Polina Zavershinskaia

**Affiliations:** Institute for Political Science, Leipzig University, Beethovenstraße 15, 04107 Leipzig, Germany

**Keywords:** Right-wing populism, Civil sphere theory, Political narrative, Violence, Germany, Covid-19

## Abstract

This paper considers the impact of the Covid-19 pandemic on right-wing populists’ constructions of German collective identity. In their “Covid-19 crisis” narratives, German populists attempted to rearrange the discursive and institutional space of the German civil sphere through a symbolic inversion of the heroic signifier and legitimization of violence against perceived enemies. To analyze such discursive dynamics, this paper utilizes multilayered narrative analysis, drawing on the synthesis of civil sphere theory, the anthropological conceptualization of the relationship between mimetic crisis and symbolic substitution of violence and the sociological narrative theory of the sacralization and desacralization of heroism. This analysis structures the investigation of positive and negative symbolic constructions of German collective identity by German right-wing populist narratives. The analysis shows that although German right-wing populists are politically peripheral, their affective, antagonistic and anti-elite narratives contribute to the semantic erosion of the liberal democratic core of the German civil sphere. This in turn reduces the ability of democratic institutions to control violence and leads to the restriction of civil solidarity.

**Supplementary Information:**

The online version contains supplementary material available at 10.1057/s41290-023-00189-2.

## Introduction

In recent years, right-wing populist narratives have been spreading in European civil societies. Scholars associate this phenomenon, *inter alia*, with the conflict of political digital communication accompanied by citizens’ decreasing trust in the EU’s democratic institutions (e.g., Krastev and Holmes 2019) and with failures of neoliberal institutionalization (e.g., Tebaldi 2020, 2021). Alexander (2019) argues that such dynamics are marked by a right-wing populist “backlash” attempting to replace the liberal democratic “frontlash” discourse. More specifically, in Germany, right-wing populist actors’ (RPAs) construction of collective identity is characterized by their attempts to symbolically delegitimize the liberal democratic discursive core and the institutions of the German civil sphere (Alexander 2006, 2021). By exacerbating feelings of crisis (e.g., Moffitt 2016), populist polarizing narratives set in motion a value-normative inversion of civil discourses.

Although right-wing populism is an attribute of the civil sphere and has commonalities with the communicative practices of everyday symbolic coding of center and left-wing populism (Alexander 2021), it has a significant destructive semantic potential in relation to the symbolic structures of inclusive civil solidarity. This can be explained by the fact that right-wing populist discourse is more closely related to the basic structures of “banality” (Wodak 2015; see also Billig 1995) and its construction of exclusionary ethnic, racial, or religious barriers. Therefore, right-wing populist discourse is a powerful trigger for the uncontrolled growth of differences between social actors and the desacralization of more complex practices of civil competition.

Furthermore, right-wing populist discourse contributes to a significant increase in violent behavior patterns. This is also expressed in the promiscuity of right-wing actors in choosing situational allies due to populists’ willingness to grant them the right to use violence. For instance, right-wing populists frequently attend far-right and extremist protests (see, e.g., Eckersley 2021; Heinze and Weisskircher 2022), “pushing the limits of what is considered civil in Germany” (Stack and Alexander 2021, p. 4) and even aggravating breaches of civil order.

Right-wing populist narratives systematically cultivate exclusionary semantic barriers to limit liberal interpretations of human rights within the community and in relation to citizens of other countries in the name of preserving “people’s democracy” and “folk culture.” Even while appealing to individual freedoms, as will be observed below in the example of the “Covid-19 crisis” narrative, right-wing populists ultimately focus not on individual self-sacrifice for the sake of others but require collective sacrifice, producing new and increasingly numerous “scapegoats.” Specifically, right-wing populists radically invert narrative structures of “heroism” and “perpetration,” which transforms the existing positive practices of “substitutive violence” (Girard 1979 [1972]) into violent practices of negative anticivil identification.

This transformation of agonistic manifestations of the civil sphere into the antagonism of total anticivil intransigence is characteristic of populist discourse more generally (Alexander et al. 2021). In this regard, the spread and consolidation of right-wing populist discourses stimulates the polarizing conflict of civil and anticivil patterns in German collective identity, expressed in the antagonistic “us against the center.” Herewith, the populist discourse “us against them,” as well as the polarizing narratives that constitute it, goes beyond positive public criticism putatively attacking the elites but in fact targeting the discursive center of the civil sphere and the organizational autonomy of its communicative and regulative institutions (Alexander 2021). The crisis of liberal political discourses and the growing relevance of previously peripheral right-wing populist narratives imitating solidarity leads to a deepening of the value-normative polarization of the civil sphere, which in turn makes civil competition radical and increasingly violent.

In the German civil sphere, such polarizing narration is characteristic of several significant right-wing populist actors (RPAs): Alternative for Germany (AfD), Patriotic Europeans Against the Islamization of the West (Pegida) and the *Identitäre Bewegung Deutschland* (German Identitarian Movement; IBD) 1. These groups, the first a registered political party and the latter two social movements, form the focus of the analysis offered here based on the premise that they produce the symbolic components of the German right-wing populist discourse.

The period studied ranges from the moment of the “Covid-19 crisis” discursive construction and symbolic representation by the chosen RPAs in March 2020, through until January 2022^2^. This period was chosen because of the ambivalent impact of the Covid-19 pandemic on the discursive space of the German civil sphere. On the one hand, the pandemic expanded the space of solidarity in the face of a common threat, while on the other hand, as evidenced by right-wing populist narration, it foregrounded the construction of negative practices of heroization and perpetration expressed in the search for ever new internal scapegoats among democratic civil actors and the legitimization of violence against them^3^.

On the basis of these data, the paper attempts to answer the following questions: What are the genre specifics of right-wing populist narratives and their symbolic components in the civil sphere? What is the performative potential of right-wing populist narratives in the construction/deconstruction of the symbolic structures of the civil sphere and collective identity? What is the uptake of such right-wing populist narratives by German civil society and its actors?

To answer these questions, I will conduct a multilayered narrative analysis (MNA). This relies on a synthesis of Alexander‘s civil sphere theory (CST), Girard’s (1979 [1972], 2016 [1978]) concept of “substitute violence,” Giesen’s (2004) analysis of symbolic figurations of political narratives and Smith’s (2005) description of narrative genres. Drawing on Girard’s conceptualization of “positive” and “negative” mimesis together with Giesen’s analysis of sacralization and desacralization of heroism and perpetration allows me to analyze the binary dynamics of RPAs’ narratives and their effects on the civil sphere^4^.

Through MNA, I examine the symbolic representations in right-wing populist narratives and consider their effects on civil discourses and the institutions that control violence in the civil sphere. MNA is composed of two stages: (1) binaries and (2) narrative genres. The first stage is devoted to the depiction and examination of right-wing populist narratives, their symbolic structures, and their influence on the discursive dynamics of the German civil sphere. The second stage is focused on identifying the genre specifics of the right-wing populist narration in the German civil sphere and its influence on the discursive structures of the civil sphere.

Following this MNA, I argue that simulative narratives of a “true” democratic order constitute the right-wing populist discursive core and destroy the existing symbolic solidarity of German civil society. RPAs’ narratives delegitimize their opponents and lionize violence against them through the semantic inversion of the sacred figures of the civil and the profane figures of the anticivil, shown in the symbolically inverted figures of the “hero” and “perpetrator.” Such symbolic strategies may change the dominant symbolic meaning of the existing civil sphere discourses, which rest on the narration of heroic sacrifice that resists the chaos of violence and expands the space for democratic inclusion (Alexander 2021; Binder 2021).

The first section of this paper details the theoretical framework; specifically, the CST employed in the analysis of right-wing populist narratives. Drawing on MNA, in the following empirical section, I answer the three abovementioned questions by analyzing the construction of German collective identity by the AfD, Pegida, and IBD amidst the Covid-19 pandemic. After I discuss the public reaction to and the influence of right-wing populist narration in the German civil sphere, using information from German communicative institutions. Finally, in the discussion, I summarize my findings and present the prospects for future research.

## Theoretical framework

When studying populist identity construction, I consider the phenomenon of collective identity within a cultural-sociological framework, where cultural processes are interpreted as somewhat independent from political and economic interests and structures (Alexander and Smith 2006). Specifically, following a cultural-sociological approach (e.g., Alexander 2006; Giesen 2004; see also Olick 2016), collective identity is understood as a set of collective representations and ritual practices of solidarity and its symbolic discursive components^5^ are considered as “independent variables” (Alexander and Smith 2006). Such an approach can explain why political actors prefer to invest extensive resources in symbolic politics (Olick 2016; see also Simko and Olick 2021) and how cultural specifics influence their strategic narratives. In this section, I will first introduce the cultural-sociological CST combining it with the abovementioned theories of Girard and Giesen, which I use to describe the alternative construction of collective identity by right-wing populists. I will then detail the theoretical description of RPAs’ symbolic performance in the civil sphere and genres of political narration before addressing specifically RPAs’ constructions of German collective identity amidst the Covid-19 pandemic.

### The civil sphere concept and the narrative analysis of its symbolic figurations

The cultural-sociological theoretical basis for this study is the concept of the “civil sphere” proposed by Alexander, who defines it as a “space for democracy” and “the arena of collective solidarity in universalistic terms” (2006, p. 43). Alexander further suggests that the symbolic codes and narratives of solidarity that underlie the discourses of the civil sphere can provide a collective identification uniting people of different classes, races, religions, and ethnicities.

The civil sphere is discursively constituted through a narrative structure centered on the binary between “sacred” and “profane,” “pure” and “impure.” Narratives order binaries through sequencing (Ricoeur 1984, cited in Smith 2005, p. 18) in order to provide answers to the “ultimate” questions of collective existence and morality, align people’s actions with such answers and mobilize powerful emotions (e.g., anxiety, fear, hatred, reverence), which influence the construction not only of the cognitive and moral symbolic dimensions of the collective identity but also expressive and affective ones. Alexander (2006) argues that ritual practices of maintaining social solidarity in any society are organized by assigning symbols of “sacred purity” to the significance of collective existence and the “profane” to the danger of the destruction of solidarity. However, while they share a basic structure and solidaristic ideal, narratives of the civil sphere differ between communities depending on the evolution of its basic discursive structures and solidarity codes.

The concept of the civil sphere is complementary to social anthropology’s (Girard 1979 [1972], 2016 [1978]) and cultural sociology’s (Giesen 2004) theorization of solidarity and the symbolic control of violence. In his mimetic theory, the anthropologist Girard (1979 [1972], 2016 [1978]) suggested that the ambivalent practices of the social construction of civil solidarity are associated with the phenomenon of “substitute violence” and the related metaphor of the “search for a scapegoat”; these are employed to symbolically regulate social inclusion, restrict civil violence, and restore social reciprocity. As a rule, the intensification of the “search for a scapegoat” is associated with a traumatic social experience—a mimetic crisis of solidarity collapse and an outbreak of uncontrolled violence. The complex symbolic practices for restoring normative order in the civil sphere (Mast 2021) are designed to replace the chaos of violence. The “substitute sacrifice” restores the binaries of divine/mundane and hero/perpetrator, preventing the return of “evil-violence” to society (Alexander 2012). Consequently, the collective identification is defined through various sacralization rituals, which aim at constructing a tangible positive similarity between society’s members—“civil us”—and excluding the Other as different and incompatible with the community’s order—“anticivil them” (Eisenstadt and Giesen 1995; Alexander 2006, p. 55; see also Smith 2005; Eisenstadt and Giesen 1995; Luhmann 1982; Karolewski and Suszycki 2011).

The binary opposition of “us versus them” determines the construction of the main figures of collective memory, the “heroes,” “perpetrators,” and “victims” (Giesen 2004)—“empty signifiers” (Laclau 2005) endowed with different meanings depending on the particular community. For Giesen (2004), the central figure of collective narratives is the “triumphant hero,” who challenges conventional wisdom and the oppressing order and defends the honor of the dehumanized “victims.” The “hero” is generally attributed to the society’s members and represents the essence of the civil. The “hero” requires constant recognition and sacralization from society; without support and admiration, the figures of “heroes” grow increasingly mundane and may even transform into the figures of “villains,” “demons,” or “perpetrators” (Giesen 2004, pp. 20–21) who create chaos, exclude victims from society and destroy social solidarity. This categorization regards the figures of the “hero” and the “perpetrator” as active subjects, while the “victims,” according to Giesen, are objectified and are passive recipients of their actions. This categorization is taken as a basis for MNA detailed in this paper.

### Right-wing populism in the civil sphere

In right-wing populist narratives, democracy is represented as a derivative of the non-reflexive and non-critical “will of the people.” Accordingly, RPAs realize their desire for power through a constant search and symbolization of the “exclusion of the Other,” the violent reorganization of the world to restore the alleged justice and “the people’s order,” presenting violence as heroic, or a genuine object of desire. These are simulative narrative, shadows of civil democratic narration. If “heroes” of civil solidarity personify the overcoming of violence and the sacralization of inclusion (Alexander 2006), recreating and expanding the symbolic boundaries of the civil sphere, then right-wing populist “pseudo-heroes” are their evil twins who destroy the civil, attempt to legitimize violence and social exclusion, and discredit the civil sphere’s institutions.

The risk of a mimetic crisis is most prominent in the right-wing populist narratives. In contemporary societies marked by mimetic crises, RPAs claim a significant place and even dominance in the civil sphere, presenting themselves as the only representatives of the people’s democracy. In contrast to the agonistic semantic core of the democratic civil sphere, the populist radical antagonistic coding is aimed at desacralizing its civil center. This coding is represented through the semantic inversion of heroism and perpetration, which transforms this into the “people versus elites,” destroying civil solidarity and generating social deviations of anticivil status. Such populist polarization can destroy civil associativity, amplifying often-violent exclusion of “them” from the symbolic framework of society due to their alleged inconsistency with the physical and cultural attributes of a given society (on which see, e.g., Alexander 2020; Ostertag 2020; Khosrokhavar 2020).

By adopting the antagonistic meanings of the symbolic figures under the binary coding “us versus the center,” RPAs construct alternative collective identities and attempt to symbolically invert the civil sphere based on their discriminatory narratives. These narratives rely on the desacralization of the civil sphere and stigmatization of chosen “perpetrators” as profane anticivil, “mad and conspiratorial” elites and their “client,” the racialized Other. Additionally, such right-wing narratives are characterized by the systematic identification of the “native people” with the figure of oppressed “victims,” which is simultaneously a “sacred” category (for more details, see Arteaga Botello 2021; Binder 2021; Morgan 2021). Finally, RPAs usually attribute themselves to the “sacred” category of “heroes” in an attempt to self-legitimize themselves as representatives of the “people’s will” (on which, see Freistein and Gadinger 2019; Kaya and Tecmen 2021; Moffitt 2016; Wodak 2015). Rather than overcoming a mimetic crisis and constructing more optimal forms of competitive solidarity, RPA’s imitative competition desacralizes and antagonizes it, as RPA permanently cultivate a state of constant war by searching for new scapegoats within the community. This chaos of violence is sacralized by right-wing populist actors as the “people’s will”—an expression of the people’s self-government.

Turning to the specifics of the Covid-19 pandemic in Germany, RPAs’ negative symbolic stigmatization of the civil sphere’s center stimulated calls for violence against the German government and civil institutions in the digital space (e.g., Telegram) and during *Querdenken Demos*, leading to physical and verbal attacks against politicians, journalists, and virologists, among others. For instance, radical right-wing actors and their supporters demanded the implementation of the “people’s” court-martial against the former German chancellor Angela Merkel and the virologist Christian Drosten as a response to their suggestions regarding anti-Covid-19 measures which were framed by RPAs as characteristic of a dictatorship (Stolz 2020). Other instances of violent articulation were a murder plot against Saxony minister-president Michael Kretschmer and other members of the Saxony cabinet in the Telegram-chat of a group associated with *Querdenker* (Heinke 2022; Hartleb 2022) and the murder of a cashier by a Covid-19 denier in Idar-Oberstein, which reportedly followed the cashier’s demand to respect masks-wearing requirements (Hövermann 2021). These requirements were often framed as the “antidemocratic” measures of an “authoritarian” government in the right-wing populist narratives (see the “Covid-19 crisis” in Pegida’s narratives below).

Such instances show how positive sacralization of “heroic” sacrifice, arising from overcoming a mimetic crisis and relying on legitimate coercion and institutions of civil ritualization inherent to the liberal democratic discourse, is subjected to semantic erosion by right-wing populist simulations. Here, “doing politics” is replaced by “mimicking of politics” (Luhmann 1982); in particular, by a dangerous “mimicking [of] democracy” (Heins and Unrau 2020) leading to an increase in violence in the civil sphere.

### The typology of narrative genres

Narratives, as argued above, order binary dichotomies of codes through semantic and symbolic preference. Therefore, studying the semantics of these oppositions by analyzing the binaries of “heroism” and “perpetration” is insufficient to identify the symbolic construction of political narratives. Therefore, it seems necessary to supplement the analysis with a study of the communicative potential of competing narrative genres and semantic structures for ordering binary coding systems. Within the competitive space of the civil sphere, social actors struggle not only for the acquisition of resources and organizations, but also, through the production of certain narratives, for the establishment of their cultural dominance in the civil sphere.

During periods of social tension, the political struggle among certain actors for dominance in the civil sphere intensifies and can be resolved by the hegemony of one or another narrative about “good and evil” (Alexander 2006, p. 65, 2012). These are designed to legitimize the positions of specific sociopolitical actors and delegitimize the positions of their opponents. Scholars (Wodak 2015; see also de Cesari and Kaya 2021; Moffitt 2016; Taggart 2000) suggest that right-wing populists continuously construct a sense of crisis, provoking the fear that elites and the cultural Other and narrowing civil solidarity (Alexander 2021, p. 2). At the same time, right-wing populists desacralize democratic human rights, speaking on behalf of “impersonal” symbolic objects, such as “people” and the “majority,” creating preconditions for the conflict-producing mimesis and heroization of the perpetration.

To identify the genre specifics of the populist narratives, I utilize the typology of genre modes developed in cultural sociology (Smith 2005). Smith distinguishes three modes of political narrative genres: low mimesis, tragedy/romance, and apocalypse. The low mimetic mode is the predominant genre of everyday politics. It is suitable for describing everyday events that do not seem dramatic to recipients, who represent an “emotionally detached audience” (Smith 2005, p. 25). The tragedy/romance mode evokes intense feelings of moral empathy, pity, and horror and is characterized by strong character transformation and plot development. Motives for action in tragedy/romance are more clearly defined by the parameters of good and evil than by everyday-life routines. One can already find polarization in these genres; specifically, the division into “heroes” and “perpetrators.” The last of Smith’s three typologies is the apocalyptic genre, which, for Smith, is the most powerful of all narrative genres and facilitates the overcoming of cultural restrictions on violence and gathering of support for sacrificing priceless human lives. The apocalyptic genre also emphasizes the impossibility of compromising with “radical evil” (Smith 2005, p. 27). The present appears here as a horror scenario that symbolizes the onset of the chaos of violence. In the case of the apocalyptic narrative structure, the “crisis,” followed immediately by a mimetic crisis, must serve as a stimulus to organize an entirely new world (Unrau 2021).

## Method: multilayered narrative analysis

Drawing on the synthesis of the CST (Alexander 2006), “substitute violence” (Girard 1979 [1972], 2016 [1978]), symbolic figurations of collective memory (Giesen 2004) and narrative genres (Smith 2005) as outlined in the previous section, this study employs MNA to examine the narrative content^6^ of right-wing populist communicative institutions^7^ and their constructions of German alternative collective identity. As noted in the introduction, the analysis focuses on the textual narration of the “Covid-19 crisis” by three selected German right-wing populist actors, the AfD, Pegida and the IBD, looking at the former’s affiliated media outlet *AfD Kompakt* and at blogs on the website of the latter two.

The analysis is divided into two stages. In the first stage, I consider the binary coding of German right-wing populist narratives. This includes addressing the conceptualization of symbolic figurations regarding the interconnection between a mimetic crisis, sacralization, power, and the symbolic othering of the chosen “perpetrators” (see Table 1)^8^. Special attention is paid to the binary coding of the RPAs’ polarizing narratives; specifically, to the symbolic figures of the “hero” and the “perpetrator” and the metaphors of their actions toward “victims”—“the native people” (Wodak 2015; see also Freistein and Gadinger 2019).

**Table 1:** Binary matrix—Binary symbolic figures of the civil sphere.

Civil (C)		Anticivil (A)	
CF	Hero	AF	Perpetrator
Symbolic figures (F)			
CF1	Triumphant hero	AF1	Perpetrator-victor
CF2	Hero-savior	AF2	Perpetrator-invader
CF3	Prophetic hero	AF3	Deceitful/perverted perpetrator
CF4	Hero-martyr	AF4	Vicious perpetrator
CF5	Tragic hero	AF5	Defeated perpetrator
Permissible actions toward victims (Av)			
CAv1	Restore honor/remember	AAv1	Ignore sufferings/silence
CAv2	Save/protect	AAv2	Abuse/misuse
CAv3	Prevent/predict suffering	AAv3	Hide the truth/demonize
CAv4	Suffer for the common well-being	AAv4	Prosecute for self-interest
CAv5	Fail to prevent suffering	AAv5	Fail and cause more suffering

This table is based on Alexander’s (2006, pp. 57–59, 2010, p. 284; see also Smith 2005, pp. 15–16) binary structure of civil discourse and Giesen’s (2004) symbolic figures of collective memory. The categorization of symbolic figures (F) and the permissible actions toward victims (Av) was made according to right-wing populist self-categorization and the categorization of their “enemies”—“the elites” and the “nefarious Other” presented in studies on right-wing populism (e.g., Brubaker 2018; Freistein and Gadinger 2019; Kaya and Tecmen 2021; Mudde 2017; Snow and Bernatzky 2018; Pelinka 2013; Wodak 2015).

**Fig. 1:**
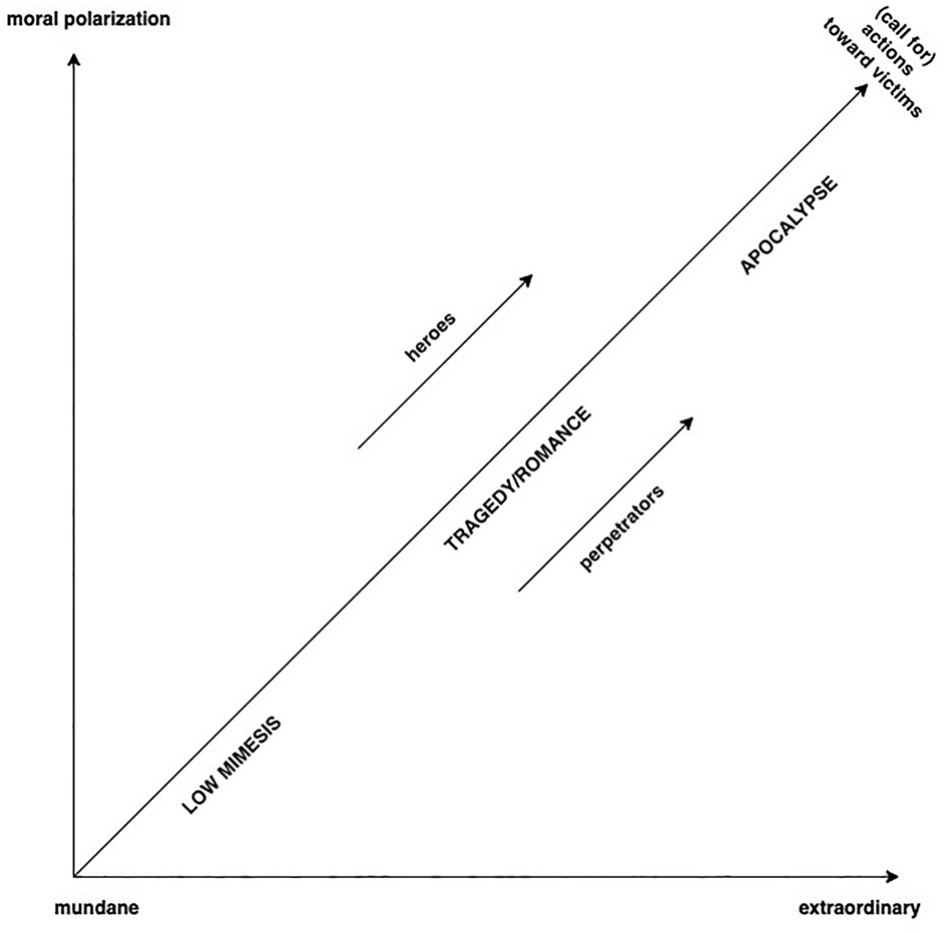
Correlation between the dynamics of symbolic figures and genre modes of civil discourse. The figure relies on the binary matrix from Table 1 and is based on Smith’s (2005, p. 24) structural model of genre and Giesen’s (2004) symbolic figures of collective memory.

In the second stage, using a narrative analysis within a cultural-sociological approach (Smith 2005), I identify the genre specifics of right-wing populist narration (see Fig. 1). This depends on the frequency of the aforementioned binary metaphors in the selected right-wing populist narrative texts.

## Empirical analysis

### The right-wing populist construction of “Covid-19 crisis” in Germany

The narrative of “'true' democracy of the people versus the 'imitative democracy' of political elites” occupies an important place in the study of populist discourses (e.g., Mudde 2017; Taggart 2000; Wodak 2015). Within this wider framework, I here focus on German RPAs’ simulation of democracy expressed in their antagonistic construction of collective identity in the so-called “Covid-19 crisis”^9^ and its association with the inversion of civil heroism and symbolization of violence.

The specifics of the “Covid-19 crisis” propagated by German RPAs was expressed in a cult of violence. This was not only directed at minority groups—specifically, the racialized and gendered Others broadly discussed in the scholarly literature (e.g., Vieten 2020; Marcks and Pawelz 2022)—but also aimed at amplifying the irreconcilable differences between the “native” people versus “antidemocratic” elites. In this way, RPAs stimulated violence toward the center of the civil sphere, expressing it in the aforementioned attacks against governmental authorities, the police and its civil institutions, *inter alia*, media and research institutes.

#### The “Covid-19 crisis” in AfD’s narratives

By homogenizing the binary differences within the “German people” and antagonizing its semantic dimension through the narrative construct of “criminal German and EU elites,” the AfD constructed its specific counternarratives of the so-called “Covid-19 crisis,” whose main initiators were said to be the German political establishment, media structures, associations and scientific institutions (AfD Kompakt 2021a). Interestingly, the radical antagonism toward “German elites” in AfD’s narratives about the “Covid-19 crisis” almost replaced their standard antagonism toward the racialized Other, such as “illegal migrants” (Buarque and Zavershinskaia 2022; see also Eatwell 2018; Havertz 2021; Wodak 2019). On the website of the AfD-affiliated media outlet *AfD Kompakt*, the dominant antiimmigration rhetoric within the “Covid-19 crisis” was present only in the context of “the need to close borders” and suggestions to “use empty seats on planes to repatriate migrants”^10^ (AfD Kompakt 2020b).

The symbolic dynamics of AfD’s “Covid-19 crisis” narratives were characterized by a high variability of antagonistic binary coding (see Table 2). At the beginning of the pandemic, the AfD’s narration was similar to the liberal democratic narratives in German civil society. The AfD claimed that heroism was expressed in personal responsibility, self-isolation and communicating online during the pandemic’s outbreak (AfD Kompakt 2020e).

**Table 2:** Frequency of binary symbolic figures in the “Covid-19 crisis” narratives of the AfD.

Civil (C)		Anticivil (A)	
Figure type (hero)	Frequency in texts	Figure type (perpetrator)	Frequency in texts
CF1	17	AF1	0
CF2	19	AF2	22
CF3	10	AF3	28
CF4	3	AF4	32
CF5	0	AF5	33

Action type (hero)	Frequency in texts	Action type (perpetrator)	Frequency in texts
CAv1	43	AAv1	4
CAv2	17	AAv2	44
CAv3	47	AAv3	37
CAv4	3	AAv4	38
CAv5	0	AAv5	39

This table is based on 92 narrative texts chosen by random sampling from March 2020 until January 2022 (total amount = 1140 narrative texts) from *AfD Kompakt*—the official media outlet of the AfD. For additional information regarding figures, see Table 1.

However, a few weeks after the official implementation of lockdowns in Germany, such narratives were replaced by more antagonistic and discriminatory constructions toward “German elites”: the “heroes” were allegedly not those who stayed home, following the requirements established by legal institutions, but those took to the streets and protested against the alleged infringement of freedoms through lockdowns (AfD Kompakt 2020d). As mentioned above, such protests against the German government’s Covid-19 restrictions often led to verbal and physical violence against the center of the German civil sphere, targeting its institutions and government figures, in turn causing violent clashes with counter-protestors (MDR 2020). In the following, I will outline the various figures implicated in AfD’s “Covid-19 crisis” narratives.

##### Heroes

The most common protagonist figures in AfD’s narratives about the “Covid-19 crisis” were the “hero-savior” and “triumphant hero.” The latter was attributed to both the “people” and the party itself as an inseparable part of the “people.” Interestingly, the future tense was used by the AfD to convince society of a future “triumphal” success and restoration of order, which would be achieved by the AfD. Noting its supposedly irrefutable strength and confidence in the coming victory against the “sinister elites,” the AfD claimed that

*given the sinister intentions of many in power, there is no reason to bury your head in the sand*. The mobilization success, for example for the freedom demonstration next Saturday, shows that *a lot of people have woken up*. And our courts often work very well in accordance with our constitution. Most police officers, soldiers and civil servants are aware of their responsibility for our country and the rule of law and are very good at distinguishing between right and wrong. *We have the truth, the law and the love of freedom on our side and with the members of the Alternative for Germany our voice can be heard loudly in the Bundestag and in all state parliaments.* … In the end, however, *we will then have the staying power and regain national sovereignty, the rule of law and social peace for all of us—including our current political opponents.* (AfD Kompakt 2020d; emphasis added)

Strikingly, despite opposing themselves to the core of the current German civil sphere and generally antagonizing its actors, in this specific statement, the AfD seemed to support regulative institutions of the civil sphere, appealing to the rule of law and police enforcement. Such rhetoric misleadingly suggested that the AfD was fighting for the liberation of civil sphere institutions from the dictatorship of the German government. However, this statement only imitated the code of the civil sphere, undermining the ideas of voluntary isolation and individual self-sacrifice as pillars of democratic broad solidarity in the face of a common threat during the pandemic. By referring to the civil sphere’s regulative institutions as a “dictatorship of the elites,” this delegitimization of the German government’s measures to combat Covid-19 was still employed by the party to radically invert the discursive content of the civil sphere and delegitimize its civil core, including the German government, where the purpose of regulative institutions was reduced, simplified, and inverted to suit this particular RPA’s purpose. As Alexander (2021, p. 3) argues, populism represents a danger to the democratic civil sphere when its “simplistic yet inveterate binarism is employed to constrain the autonomy of the civil sphere institutions that sustain democratic life.”

The AfD, as an alleged “people’s savior” who would rescue “the people” from the so-called Covid-19 economic crisis and “irresponsible governmental politics,” demanded the return of freedoms and democracy to the “German people.” These had supposedly been taken from them by the “authoritarian” government. Accordingly, the party called for the abolition of lockdowns, restrictive measures, and vaccination policies; the return of “reason to politics” instead of the alleged panic and rash decisions (AfD Kompakt 2021c); and the “rescue” of the German economy from the perceived “authoritarian” lockdown measures and the “plundering” politics of Brussels (e.g., by the issuing Eurobonds as “Coronabonds” during the pandemic). For instance, the AfD claimed that revealing the “destructive” activity of the German government was supposedly part of its duty to protect German citizens: “if Corona-measures disproportionately interfere with the basic rights of citizens—i.e., if they are unconstitutional—the freedom of our democracy will be endangered. It is *the duty of the opposition party to point this out*” (AfD Kompakt 2020a; emphasis added).

In addition to proposing the abovementioned measures to allegedly save the “German people” from irresponsible “governmental corona politics,” the figure of the “hero-prophet” was also frequent in AfD’s narratives. Such a “hero” supposedly predicted the difficulties for the German people under the current “authoritarian” governmental politics. For instance, the AfD claimed “as AfD, we *had predicted the effects of the irresponsible corona policy very early on* and called for resolute countermeasures. The current figures clearly show that it was a mistake not to listen to us!” (AfD Kompakt 2020g; emphasis added).

While some scholars argue that right-wing populist discourse aims at RPAs’ self-victimization (Müller 2016; see also Binder 2021; Schmalenberger and Hübscher 2022), the figure of the “hero-martyr” is rarely evident in AfD’s narratives. On those occasions it was employed, the “hero” was often connected to the elites’ supposed aim to defame the name the party and to the willingness of the AfD to accept this “sacrifice” for the well-being of the “people”: “in December 2020, the AfD parliamentary group submitted an emergency motion entitled ‘End state-ordered endangerment of children’s welfare immediately’ in the state parliament. *We were severely insulted for this by the old parties*” (AfD Kompakt 2022; emphasis added). Finally, the figure of the “tragic hero,” suffering and broken by a “traumatic reality” (Giesen 2004), seemed to be generally absent.

##### Perpetrators

The primary antagonist in the AfD’s “Covid-19 crisis” was the German government, first led by Angela Merkel and then by her successor Olaf Scholz. The European Union, communicative institutions of the German civil sphere, such as media companies (e.g., ZDF), and scientific institutions (e.g., Robert Koch Institute) were also stigmatized. The AfD variously described the government’s anti-Covid-19 politics as either “panic” or “authoritarian,” often comparing it to the German Democratic Republic (GDR, East Germany) and the dystopias of George Orwell and Aldous Huxley (e.g., AfD Kompakt 2020c, 2021d).

The most common “perpetrator” type in AfD’s narratives was the “defeated perpetrator.” The AfD claimed that the anti-Covid measures of the German government were thoughtless and were dragging German society into a social and economic crisis. The AfD suggested that even when the German government’s efforts to combat the pandemic were effective, they could have supposedly been implemented much sooner:

*The federal government overdid it*. The lockdown was also inevitable because [the government] did not take the Coronavirus seriously at first and accordingly made inadequate medical preparations. *Just as it came too late, the relaxations [of regulations] came too late.* (AfD Kompakt 2020h; emphasis added)

Additionally, counter-figures of a “vicious perpetrator” and a “deceitful/perverted perpetrator” were popular in AfD’s narratives. On the one hand, the German “authoritarian/totalitarian” elites supposedly used the pandemic to restrict people’s freedom for their individual purposes (“vicious perpetrator”), as shown in the following: “after the disastrous judgment of the Federal Constitutional Court on the Corona measures, the future *SPD chancellor has dropped all his inhibitions*. On Twitter, *he uttered incredible words reminiscent of totalitarian horror books from* 1984 *to* Brave New World” (AfD Kompakt 2021d; emphasis added). On the other hand, the elites allegedly fabricated lies and excuses to cover up their criminal activities (“deceitful/perverted perpetrator”): “*with a flimsy pretext*, the Federal Minister of Health is making a *new attempt to foist his Trojan horse of an ‘immunity card’ on the public*” (AfD Kompakt 2020f; emphasis added).

The figure of the “perpetrator-invader” was used less often and usually in connection with the actions of the EU’s political apparatus, which allegedly encroached on the money of “poor” German taxpayers and gave it to “rich Spaniards and Italians” under the pretext of fighting Covid-19 (AfD Kompakt 2021b). Finally, the figure of a triumphant omnipotent “perpetrator-victor” did not seem to occur in any of the analyzed texts.

##### Constructing narratives

Drawing on binary division and its symbolic figurations, AfD’s “Covid-19 crisis” narratives can be summarized as follows: During the coronavirus pandemic, the autocratic politics of the German and European elites aimed at restricting the democratic freedoms of the German people, which led to a severe economic crisis and the problematic psychological state of German citizens. Even when the German elites could rectify the situation, they panicked and were extremely incompetent in their actions, which exacerbated the crisis. To prevent the German people from noticing the elite’s failed policies and self-interest in seeking to achieve absolute power, they hid the truth and fed German society with lies. As a result, the present has turned into a disaster, and the future of Germany looks bleak unless the AfD, with the support of the German people, restores the democratic order and puts an end to the chaos of the elites.

Overall, AfD’s narratives about the “Covid-19 crisis” relied on the antagonism of the “German democratic people” toward the “German and European autocratic elites.” The symbolic dynamics of AfD’s narratives indicated the romantic/tragic genre with some elements of low mimesis and apocalypse. Despite the noticeably frequent use of the apocalyptic metaphors “catastrophe,” “panic” and “madness,” the AfD primarily alleged a “panic caused by inaction,” which was not the fault of an omnipotent perpetrator, an absolute evil that is inherent for the apocalyptic genre (Smith 2005), but was about supposedly incompetent political elites who, due to their stupidity or greed, provoked a “crisis.” According to the AfD, to preserve or revive the old “normal” order (*alte Normalität zurück*), “German and European elites” had to be “put in their place” otherwise “corona panic” could destroy German society (AfD Kompakt 2021e). Thus, the AfD’s construction of collective identity was expressed in a combination of nostalgia for the great past and pessimistic notions about the present and future (Binder 2021, p. 182).

Finally, despite the absence of open calls for violence, such antagonistic hybrid narratives still inspired it against the representatives of the German civil sphere and its communicative and regulative institutions. The AfD’s support for public demonstrations against the government’s Covid-19 restrictions and their delegitimization of civil institutions found vast support from the *Querdenker*-protesters, including the AfD’s members among them (Hartleb 2022), who often promoted violence against the core of the German civil sphere.

#### The “Covid-19 crisis” in Pegida’s narratives

Regularly recurring street demonstrations had been the defining feature of Pegida’s protest movement before the Covid-19 pandemic. However, starting from the end of March 2020, due to the lockdowns and restrictive anti-Covid-19 measures in Germany, Pegida had to change its activities to a “virtual form of protest” (Volk 2021), expressed mainly through blogging on Pegida’s website and YouTube broadcasts, including digital versions of their demonstrations.

Pegida’s official website blog was chosen as the source for my analysis. The symbolic activity of Pegida and its construction of German collective identity during the Covid-19 pandemic was represented by a relatively smaller number of blog publications about the “Covid-19 crisis” than those presented on the website of *AfD Kompakt* (23 against 1140 narrative texts). This can be explained by Pegida’s preference to use social media (e.g., Telegram, YouTube) to mainstream its antagonistic narratives (Puschmann et al. 2020). Nevertheless, when conducting MNA, I could identify somewhat pronounced narratives of the “Covid-19 crisis” and their binary components (see Table 3) in Pegida’s texts.

**Table 3:** Frequency of binary symbolic figures in the “Covid-19 crisis” narratives of Pegida

Civil (C)		Anticivil (A)	
Figure type (hero)	Frequency in texts	Figure type (perpetrator)	Frequency in texts
CF1	5	AF1	0
CF2	0	AF2	0
CF3	0	AF3	1
CF4	3	AF4	6
CF5	0	AF5	2

Action type (hero)	Frequency in texts	Action type (perpetrator)	Frequency in texts
CAv1	2	AAv1	0
CAv2	0	AAv2	2
CAv3	2	AAv3	6
CAv4	2	AAv4	12
CAv5	0	AAv5	5

This table is based on 23 narrative texts within a time frame from March 2020 until January 2022 from Pegida’s official website http://www.pegida.de. For additional information regarding figures, see Table 1.

The construction of German collective identity in Pegida’s narratives of the “Covid-19 crisis” was similar to the AfD’s identity construction in the abovementioned narratives. Akin to the narratives on *AfD Kompakt*, Pegida’s narration was characterized by the pronounced antagonistic opposition between “German native people” and “autocratic elites.” Specifically, as Volk (2021, p. 244) argues, during the Covid-19 pandemic, Pegida articulated narratives in which it represented itself and the “German people” as “victims” of an alleged elite conspiracy against “the people.” For instance, Pegida claimed that measures to combat Covid-19 (e.g., medical masks) introduced by the German government were aimed at silencing “patriots.”

##### Heroes

The only categories of “hero” in Pegida’s narratives of the “Covid-19 crisis” were the “triumphant hero”—which, similarly to the AfD’s framing, were often narrated in a future tense—and, less frequently, the “hero-martyr.” On the one hand, such heroic narratives claim that Pegida members continued their “rightful” cause by protesting even at home against the “arbitrariness” of the government and “dictatorial” anti-Covid measures (e.g., lockdowns): “*PEGIDA cannot be dismissed, we stand still and use this stone*, which is placed in our way again, *to expand our lighthouse*, which radiates from Dresden over our entire homeland” (Pegida 2020a; emphasis added). On the other hand, Pegida’s demonstrators were presented as “hero-martyrs” who allegedly fought for the “freedoms” of the “German people” even though the German political and media “elites” constantly oppressed their human rights. Here we can observe the self-victimization inherent to the right-wing populist discourse (Müller 2016; see also Binder 2021; Schmalenberger and Hübscher 2022): “then as now, *you can tease, make them look like fools, humiliate and bring the people to their knees, but eventually, they will rise and hold accountable those who sought to destroy them*” (Pegida 2021a; emphasis added).

##### Perpetrators

Pegida’s narratives about the “Covid-19 crisis,” similarly to the AfD’s, introduced semantic antagonisms into the symbolic politics of the German political and media elites, desacralizing the symbolic space of civil consolidation. To Pegida, the elites used the “Coronavirus chaos” to restrict the “freedoms” of the “German people” and establish a “dictatorship.”

Accordingly, the most common counter-figure in their narratives was the “vicious perpetrator.” Similar to the AfD, Pegida claimed that the German government did not perform politics “for the people”; on the contrary, “Corona-measures” were supposedly implemented by the government to restrict the freedoms of the German “people” and to accrue more power: *this politics is not a politics for free people. Democracy and civil rights have been abolished*. Everywhere people are taking to the streets *against this politics of destruction* (Pegida 2021b; emphasis added).

The symbolic figure of the “defeated perpetrator” in the form of the “irresponsible politician” was also directed at the German “elites.” Pegida claimed that their members would reveal the “criminal” plans of the “elites” and would inform the people, thereby “expos[ing] the lies and failures of the government related to Corona” (Pegida 2020c). Additionally, in Pegida’s narratives, the figure of the “defeated perpetrator” often intersected with that of the “deceitful/perverted perpetrator”—also attributed to “German elites” who allegedly fraudulently constructed the “media virus” and distracted the “German people” from supposed fundamental problems; for instance, from a perceived democratic decline and an alleged dictatorship of the elites (Pegida 2020b, 2021a):

CORONA, CORONA, CORONA. Still *exclusively a media virus!* … *The government panic team has done a great job. Within a weekend, an entire nation was trained like a circus horse*: keep a distance of 2m, only two people are allowed on the street and only with a good reason. *People dance around each other like marionettes* with magnets of the same polarity. Well done Berlin! (Pegida 2020b; emphasis added)

##### Constructing narratives

Drawing on the abovementioned antagonistic metaphors, I identified the narratives of the “Covid-19 crisis” that support Pegida’s construction of German collective identity. These narratives can be generalized as follows: During the Covid-19 pandemic, under the pretext of protecting citizens against Covid-19 and on the basis of other lies, the German government tried to undermine democracy and impose a dictatorship. Panicked, incompetent measures to combat Covid-19, such as the cancelation of Pegida’s peaceful demonstrations, were exploited by the German government, which led to a democratic decline in Germany. However, the members of Pegida did not give up and continued their real and virtual fight for the rights of the German people.

Similar to the AfD’s narratives about the “Covid-19 crisis,” Pegida’s narratives were also characterized by the romantic/tragic genre. Some of Pegida’s blog posts were marked by positive expectations of a “better future” and belief “in the triumph of justice" inherent to the romantic mode (Smith 2005). Pegida claimed that although the German government was trying to undermine democracy through anti-Covid measures, Pegida constituted a serious democratic rebuff (e.g., Pegida 2020a, 2021a). Nevertheless, some of Pegida’s narratives were also marked by an apocalyptic narration, a reference to the perceived “chaos” created due to the “panicked” anti-Covid politics of the “German elites” and their desire for absolute power.

Such mimetic differentiation was essentially aimed at destroying the boundaries between “positive” and “negative” ideas about democracy and asserting control over dominant discourses in the civil sphere by deconstructing its solidarity narratives. This was expressed in the escalation of violent actions against the German civil sphere in the form of the abovementioned protests and in the targeting of German civil actors who allegedly threatened Pegida’s democratic order. For instance, vaccination teams were subjected to such threats by Pegida members, among others (Goertz 2022).

#### The IBD’s narration regarding Covid-19

The IBD is a Pegida-allied movement, whose street performativity (e.g., Vorländer et al 2018; Havertz 2021) before and during the Covid-19 pandemic was expressed in “protest ritualization” (Schmalenberger 2020). For instance, IBD supporters organized street flash mobs to express anti-government sentiment and participated in *Querdenken Demos*. These were presumably utilized for indoctrination and radicalization purposes (Stolz 2020) and were later documented on the IBD website’s blog.

The IBD’s utilization of the “Covid-19 crisis” to construct its version of German collective identity was rarely identified in the blog articles from March 2020 until January 2022^11^. Only a few sentences regarding “Covid-19” as a “constructed crisis” were found in their apocalyptic narration regarding the “migration crisis” and the “crisis of German democratic society,” which were associated, according to the IBD, with the “authoritarianism” of the “elites.” While articulating the issue of the “migration crisis,” the IBD argued that by *constructing* the “Covid-19 crisis,” the government overlooked and silenced the ongoing “migration crisis,” allegedly associated with “illegal immigration” from Africa and Central Asia through Turkey:

While the *Corona issue is currently overshadowing everything, a crisis is brewing in the slipstream that has almost been forgotten*. In February of this year, Turkey announced that it would send hundreds of thousands of migrants to the Greek border, effectively ending the “refugee agreement” with the European Union. *A new wave of immigration that would be rolling towards Germany and Europe on an even larger scale than in 2015*. (Identitäre Bewegung 2020a; emphasis added)

In the case of the other narrative about the “crisis of German democratic society,” the IBD drew attention to the perceived “repression” and “censorship” of “peaceful protesters” during *Querdenken Demos*, noting that “the resistant milieu had recently been confronted primarily with Corona, repression and censorship” (e.g., Identitäre Bewegung 2020b). Additionally, the IBD claimed that the government’s emphasis on the “Covid-19 crisis” supposedly distracted from the “left-wing extremism” problem:

Large parts of society agree that the Corona crisis was THE defining event and will influence the future. The constant media coverage of this topic bears witness to this focus. Other topics were pushed to the background and will probably only be assigned a supporting role in the annual reviews. *But 2020 was also the year in which violence from the left and persecution of the patriotic opposition reached a new level.* (e.g., Identitäre Bewegung 2020c; emphasis added)

Although the Covid-19 pandemic was not significant enough for the IBD to construct an antagonistic German collective identity upon, the analysis showed the utilization of Covid-19 by the IBD to desacralize the center of the German civil sphere in way that was similar to that observed in AfD’s and Pegida’s narration. For instance, the IBD attributed the construction of the “Covid-19 crisis” to the German government, which allegedly wanted to distract German society from supposedly more pressing, “real” crises. Even though such narration was not frequent on the IBD’s blog, when present, it was aimed at delegitimizing the core civil sphere actors and institutions and destroying broad solidarity by allowing and even celebrating physical violence against opponents such as the German government and supposed “left radicals.”

### Right-wing populist inversion in the German civil sphere

When studying the symbolic activity of RPAs in the German civil sphere, and to answer my third question, I consider it necessary not only to examine their construction of an alternative identity and solidarity but also to evaluate how it was received by the “lifeworld of public opinion” which links communicative and regulative institutions (Alexander 2006). In short, the effectiveness of RPAs’ symbolic struggle to dominate the German civil sphere can be evaluated as relatively low. According to scholars (e.g., Heins and Unrau 2020), even as immigration to European countries increased after 2015, Pegida’s overall German approval was not high, and their practices in the German civil sphere were relatively ineffective. RPAs’ narratives were being overshadowed by the dominant German *Willkommenskultur* (welcome culture; the narrative of a culture of acceptance toward the Other).

Despite its nature as a “traumatic event,” the Covid-19 pandemic also did not contribute to an increase in the approval of RPAs in the German civil sphere. From March 2020 until January 2022, the narrative of heroic compliance with the government’s anti-Covid regulations and condemnation of the *Querdenken Demos* as frequented by right-wing extremists (e.g., Deutsche Welle 2021; von Lieben 2021) dominated the German popular media (e.g., the broadcasters ZDF and ARD; the radio station *Deutschlandfunk*; the newspapers *Zeit*, *Spiegel*, *Frankfurter Allgemeine Zeitung*, *Deutsche Welle*) as protectors of “a community of solidarity against incursions from non-civil spheres” (Luengo and Ihlebæk 2020, p. 129).

The activity of the Pegida “cultural centers” in Dresden and the IBD in Halle (Havertz 2021, p. 102) and the dominance of the AfD in certain eastern German states (Saxony, Thuringia) were often considered by scholars as local and explained by the specifics of collective memory in the reunified Germany. This was associated with the postwar construction of a collective identity in West Germany based on temporal and spatial antagonisms “the (Nazi) past vs. the (democratic) present and the (free) West vs. the (unfree) East” (Heins and Unrau 2020, p. 147). Scholars associate the prevalence of liberal tendencies in the German civil sphere over RPAs’ antagonistic symbolic dynamics with the phenomenon of German collective memory—a so-called “collective guilt” for the “polluted” Nazi past. As Heins and Unrau emphasize, “one of the long-term consequences of the acceptance of historical guilt is the widespread attitude to side with the former victims of Nazi Germany and with the non-German Other in general” (2020, p. 145). This may explain the acceptance of the Other and the cultivation of the ideals of liberal democracy, such as broad solidarity, in contemporary Germany.

Such dynamics, at first glance, indicate that the symbolic activity of RPAs cannot be characterized by a stable growth of their influence in the German civil sphere. At the same time, I argue that right-wing populist narratives are characterized by the ability to permanently produce discriminatory antagonistic semantic differences which are marked by a kind of negative “ontological indifference” to the negative violent practices of civil sacralization. Such patterns delegitimize the existing democratic institutions in the civil sphere by desacralizing the core discourses of the collective responsibility at the heart of postwar liberal consensus (e.g., Olick 2016). Instead of supporting examples of civil heroic sacrifice in the name of collective solidarity, right-wing populist narratives offer ever-more-dangerous violent “symbolic placebos” by drawing people in search of more and new “scapegoats.”

## Conclusion

Synthesizing the CST with Girard’s anthropological premise of “substitute violence” regarding the relationship between a “mimetic crisis,” violence, the sacred and power, Giesen’s symbolic figurations of collective memory and Smith’s narrative genres, I have conducted MNA of the discursive construction of German collective identity by German RPAs during the Covid-19 pandemic and examined the effectiveness of such narratives in the German civil sphere. Based on this analysis, I have drawn several conclusions about the influence of the symbolic practices of RPAs on the discursive and institutional dynamics of the German civil sphere.

First, the right-wing populist performative construction of German collective identity draws on the inversed discursive practices of “substitutive violence” that stimulate a mimetic crisis—an imbalance of positive and negative patterns of civil behavior and civil construction, transforming the competitiveness characteristic of the civil sphere into violent actions aimed at destroying its institutions. The reconstruction of the discursive space of the German civil sphere by RPAs within the framework of their “crisis” narratives is carried out through the inversion of the symbolism of heroic civil consciousness and the reduction of a broad civil solidarity to the narrow definition of the “German people’s democracy.” If the dominant narratives of civil solidarity in Germany arise on the basis of the sacralization of heroic sacrifice, orienting toward behavioral patterns that constrain violent ways of radically challenging the political order, right-wing populist narratives symbolically destroy the distinction between polluted and sacred civil sacrifice. The spread of such narratives leads to the restriction of the civil sphere’s solidarity. Right-wing populist narratives are characterized by simulativeness, represented as a non-reflexive and non-critical “will of the people,” opposing “criminal elites.” This is accompanied by a permanent articulation of cultural boundaries leading to the desacralization and delegitimization of democratic institutions.

The second important conclusion concerns the specifics of the narrative dynamics and effectiveness of right-wing populist narratives during the Covid-19 pandemic. During the period analyzed, the public discourse of civil solidarity “in the face of collective danger” did not lead to a significant intensification of right-wing populist narratives, despite the symbolic activity of the AfD, Pegida and the IBD, realized, *inter alia*, in genre hybridization. However, the RPAs’ “Covid-19 crisis” narratives extended the “true people’s democracy” discourse expressed in their amplification of irresolvable differences between “the democratic people” and “authoritarian elites”—the “simulative twins” of democratic order. I conclude that through the permanent production of the symbolic figures of scapegoats in their “Covid-19 crisis” narratives, RPAs stimulated a value-normative inversion of civil discourses. Such narratives aim to change existing legal practices of the symbolic “substitution of violence” to narratives that expand arbitrary violence and quasi-democratic patterns of collective identification.

Finally, I argue that the general discriminatory orientation of German RPAs catalyzes the permanent diversification of the dominant narratives of civil solidarity and the delegitimization of the symbolic content of the German civil sphere. Moreover, the effective spread of such discursive practices across various platforms consistently legitimizes illegal coercion and violence in that civil sphere. In the context of deepening geopolitical fracture, inversive dynamics of national memories and the amplification of iconic symbolic content in the network structures of contemporary communicative institutions, such semantic inversion of right-wing populist narratives is a serious symbolic prerequisite for the emergence of a large, unifying right-wing populist discourse that can destroy the existing agonal discourses of the civil sphere. In this connection, a promising direction for further research would be the study of the performative specifics and the growing influence of the iconic content of right-wing populist narratives in the social construction of German national identity and institutions of civil self-organization.

## Supplementary Information

Below is the link to the electronic supplementary material.

Supplementary file1 (XLSX 110 kb)

## Data Availability

The author confirms that the data supporting the findings of this study are available within the article and its supplementary materials.

## Footnotes

1 The general criteria for selecting actors were the definition of an actor as a right-wing populist (e.g., Brubaker 2018; Wodak 2015; Havertz 2021); the description of such organizations on the *Bundesamt für Verfassungsschutz* (Federal Intelligence Service) website as *Rechtsextremistische Verdachtsfälle* (right-wing extremist suspected cases; BfV no date); that they were currently active as of February 2022; the legality of an actor within Germany; and the presence of a website. In the case of the AfD, the selected political party had seats in the German Bundestag and had received significant public funding based on the criteria of the “degree of a party’s rootedness in society” (Deutscher Bundestag 2020), judged through electoral success and receiving donations from individuals. In the case of Pegida and the IBD, the sampling of social movements was carried out according to the following criteria: “by way of search engines” (Mosca 2014, p. 402; i.e., the highest rankings according to Google page ranking using CheckPageRank) and the highest number of followers on social media (YouTube and Telegram, since the Twitter and Facebook accounts of many right-wing actors are suspended).

2 The sample was intentionally limited to the timeframe March 2020 until January 2022 to analyze a mimetic crisis and symbolic response of RPAs caused by the Covid-19 pandemic. Narrative texts from February 2022 are not considered in the sample as they may contribute to other polarizing narratives of RPAs caused by the Russian invasion of Ukraine on 24 February 2023 and the German political *Zeitenwende* (turn of an era) in response.

3 For instance, symbolic legitimization and representation of violence can be observed in the form of *Querdenken Demo(s)* (lateral thinking demonstration[s]) in Germany—demonstrations against government measures to combat Covid-19, supported by German right-wing populists as an act of alleged civil disobedience to the “authoritarian” restrictions of the German government “targeting German democracy.” Such protests often led to physical and verbal attacks against the German government, police and media (Groppe 2021).

4 The mimetic conception is used in this paper in a broader theoretical scope and is not limited to Girard’s (2016 [1978]) theological elaborations. The mimetic theory relies on the anthropological hypothesis of the relationship between a “mimetic crisis,” violence, the sacred and power (Girard 1979 [1972], 2016 [1978]), which is implemented to conceptualize social performance in the civil sphere. Mimesis as a process of “imitation and appropriation” can be interpreted as an analog of the ambivalent (“positive” and “negative”) symbolic production of behavioral patterns, leading to the construction of an imitative model of the Other. The semantic core of such imitation is associated with the cultural practices of coding violence, its symbolic channeling and substitution (social construction of a scapegoat) for symbolic transformation when the community’s existence is threatened by the competitive desacralizing activity of social actors and state institutions. Girard argued that a mimetic crisis arises in connection with the imbalance of behavioral patterns and actions and the spontaneous and uncontrolled growth of differences between social actors, leading to violence and self-destruction. Social competitiveness in this case acquires a radical, violent character, destroying structures of social institutions and threatening the existence of society. Violence loses institutional and symbolic restrictions, involving more and more members of the community in cultivating increasingly negative violent patterns leading to a “violence of indifference.” A mimetic crisis leads to the desacralization of previously existing unifying patterns of “heroism” and “perpetration,” the destruction of the institutions which restrain violence and asynchronization of communicative continuity (Giesen 2004). According to Girard, a mimetic crisis can be resolved by a collective sacrifice through the sacralization of a scapegoat (an example of the sacrificial Other), which has the symbolic potential of “positive mimesis,” involving Others in the process of virtuous coexistence. Or, on the contrary, a mimetic crisis can be deepened if people begin to worship “idols,” which ultimately leads to the desacralization of solidarity patterns and social institutions, the search for ever new scapegoats, denying some members of society the right to a solidaristic existence and sacralizing the cult of violence. I believe that such an interpretation of Girard’s mimesis is complementary to CTS, emphasizing that “democratic life depends less on voting procedures and legal rights—in and of themselves—than on the social existence of a performatively compelling, emotionally vital and morally universalizing sphere of solidarity” (Alexander 2021, p. 2). The creation and destruction of such solidarity and its institutions “depend on connecting the sacred-democratic and profane-authoritarian sides of democratic discourse to ongoing events and struggles in particular times and places” (Alexander 2021, p. 3).

5 This paper emphasizes the “ontological indifference” of social narration (Koschorke 2012) and its tolerance regarding the issues of truth/falsehood, subjectivity/objectivity, etc. Cultural sociologists (e.g., Alexander and Smith 2006; Alexander 2006; Olick 2016; Simko and Olick 2021) understand narratives as “metacodes”—cultural universals that support and create “transcultural” collective representations, overcoming the limitations of local group identities and linking together individual storytelling about the social significance of certain events.

6 The narrative analysis in this paper concerns the investigation of narrative texts as defined in social and cultural studies: both conventional narratives in blogs on websites and “small stories” (Georgakopoulou 2015) or micro-oriented analysis, which “includes statements by politicians and ordinary actors in the press by examining quotes in newspapers, YouTube videos, tweets and other social media outlets” (Gadinger et al 2019, p. 97). Here, I limited the study to the analysis of the chosen RPAs’ official blogs.

7 This paper examines the narrative content of the civil sphere’s communicative institutions; specifically, factual media (see Alexander 2006, for more details), which, according to Alexander (2006, p. 70), specialize in communicative rather than regulative tasks. The factual media include, for example, the mass media, public opinion polls, and public organizations.

8 Here, I relied on the binary matrix of civil discourse (Table 1) and utilized the mixed-methods software MAXQDA to code the defined binary elements and metaphors in the selected narrative texts.

9 The analysis encompasses 121 texts from the chosen RPAs beginning from the official start of the Covid-19 pandemic as announced by the World Health Organization (WHO)—i.e., March 2020—through January 2022.

10 Here and below, the translations from German are mine.

11 All mentions of “Covid-19” on the blog were limited to 2020. This is similar to Pegida and can be explained by the IBD’s use of alternative media (e.g., Telegram), the analysis of which falls outside the scope of this paper.
